# Preliminary assessment of biodistribution and targeting of the fluorescent molecular probe Cy7-SYL3C in an EpCAM-positive colorectal cancer mouse model

**DOI:** 10.1038/s41598-026-37787-2

**Published:** 2026-01-29

**Authors:** Yiwen Li, Min Li, Pinghui Li, Zhidie Huang, Xiang Liu, Xiaoyan Duan, Jianbo Li

**Affiliations:** 1 Inner Mongolia Medical University, Hohhot, China; 2 The Affiliated Hospital of Inner Mongolia Medical University, Hohhot, China; 3 Inner Mongolia Autonomous Region Key Laboratory of Nuclear Medicine and Molecular Imaging, Hohhot, China

**Keywords:** Aptamer, Near-infrared fluorescence imaging, Colorectal cancer, Epithelial cell adhesion molecule, Pharmacokinetics, Biological techniques, Cancer, Oncology

## Abstract

Molecular imaging probes targeting the epithelial cell adhesion molecule (EpCAM) hold considerable promise in advancing colorectal cancer (CRC) research. Building on previous work, this study further evaluated the biodistribution of Cy7-SYL3C in healthy mice and its targeting efficacy in HT-29 colorectal cancer models, confirming its potential as a near-infrared fluorescent (NIRF) imaging probe. The fluorescent molecular probe Cy7-SYL3C was synthesized by conjugating the Cy7 fluorophore to the 5’ end of the SYL3C aptamer. Biodistribution studies were conducted in healthy mice following intravenous administration of the probe. For tumor targeting evaluation, a subcutaneous HT-29 human CRC xenograft model was established in nude mice. Tumor-bearing mice were allocated into two groups: an experimental group and a pre-blocking group. The pre-blocking group received an excess of unlabeled SYL3C aptamer prior to injection of Cy7-SYL3C. Small animal in vivo imaging technology (SAFI) was employed to monitor the biological distribution and tumor targeting ability of Cy7-SYL3C at different time points from 5 min to 48 h after injection. The expression of EpCAM in tumor tissues was analyzed by Western blot. The targeting ability of the probe was evaluated through immunofluorescence co-localization and pre-blocking protocols. Cy7-SYL3C is mainly metabolized and cleared by the liver and kidneys. Fluorescence signals can be detected at the tumor site only 5 min after injection. Quantitative analysis showed that the average fluorescence intensity (AFI) at the tumor site in the experimental group was 88.2% higher (6.4 × 10^7^ photons/s/mm^2^) compared to the pre-blocking group (3.4 × 10^7^ photons/s/mm^2^) over a 4-hour observation period. Furthermore, the experimental group displayed a moderate positive correlation (Pearson’s *r* = 0.30 ± 0.02), in contrast to the negligible correlation observed in the pre-blocking group (Pearson’s *r* = 0.05 ± 0.01). The tumor-to-muscle ratio exceeded 1.0 at six hours post-injection and peaked at 1.30 ± 0.04 photons/s/mm^2^, with target-specific signals maintained for up to eight hours. This indicates that as the probe gradually removed from normal tissues such as muscles, it achieved sustained and specific retention at the tumor site. Combined with the significant reduction in the signal caused by the pre-blocking strategy, these results consistently indicated that the accumulation of Cy7-SYL3C in the body exhibited EpCAM targeting specificity. This study was based on the EpCAM targeting strategy and further evaluated the in vivo performance of the near-infrared fluorescent probe Cy7-SYL3C. The detailed dynamic imaging results at multiple time points indicated that this probe had a clear metabolic pathway in healthy mice and demonstrated rapid, sustained and specific tumor targeting ability in the HT-29 colorectal cancer model. These characteristics collectively confirmed the clinical application potential of Cy7-SYL3C as a high-performance molecular imaging tool in colorectal cancer research.

**Supplementary Information:**

The online version contains supplementary material available at 10.1038/s41598-026-37787-2.

## Introduction

Colorectal cancer (CRC) ranks among the most prevalent and deadly malignancies worldwide. According to the latest GLOBOCAN estimates, CRC is the third most commonly diagnosed cancer and the second leading cause of cancer-related mortality worldwide, underscoring the urgent need for improved diagnostic strategies^1^. Data from the World Health Organization indicate that approximately 1.9 million new cases and 930,000 CRC-related deaths occur globally each year. Despite advancements in screening practices that have reduced mortality rates in developed countries, the incidence of CRC continues to rise in developing regions, partly attributed to the adoption of Westernized dietary patterns and the aging of populations^2^. Alarmingly, approximately 60% of patients are diagnosed with advanced-stage disease, and their 5-year survival rates are below 15%^3,4^. Although endoscopic examination remains the diagnostic gold standard, its invasive nature contributes to limited screening adherence—compliance rates remain below 50%—and poses procedural risks, such as a perforation rate ranging from 0.1% to 0.3%^5^. These factors underscore an urgent need for diagnostic modalities with high sensitivity to improve early detection and clinical outcomes in CRC.

Molecular imaging enables the noninvasive visualization of disease-associated biomolecules within living organisms. Among these techniques, near-infrared fluorescence (NIRF) imaging offers distinct advantages for tumor detection, owing to its capacity for deep tissue penetration (5–10 mm) combined with high spatiotemporal resolution (approximately 50 μm)^6,7^. Nonetheless, the successful clinical application of NIRF imaging is heavily reliant on the development of highly specific molecular probes capable of targeting relevant biomarkers with precision.

Aptamers are synthetic, single-stranded oligonucleotides selected from in vitro random sequence libraries through the SELEX process (Systematic Evolution of Ligands by EXponential Enrichment)^8,9^. They exhibit high specificity and affinity for a wide variety of targets, including proteins, cells, bacteria, and small molecules^10^, earning them the designation of “chemical antibodies”^11^. Aptamers achieve precise and selective target binding by folding into unique and intricate three-dimensional conformations^12^. Noteworthy advantages of aptamers include facile chemical modification, low immunogenicity, rapid tissue penetration, and minimal toxicity^13–15^. In recent years, numerous aptamers have been developed for tumor recognition, imaging^16^, and targeted therapy^17^. For example, the aptamer AS1411 exhibits high affinity for nucleolin on the surface of gastric cancer cells, leading to inhibition of proliferation and induction of apoptosis^18^. Some aptamers are currently undergoing Phase II clinical trials for renal cancer in the United States^19^. Collectively, aptamers represent a promising class of targeting ligands for the design and development of next-generation molecular probes.

The epithelial cell adhesion molecule (EpCAM) serves as a marker for CRC stem cells^20,21^, and is aberrantly overexpressed in more than 80% of CRC cases^22^. Its elevated expression is closely associated with tumor metastasis and drug resistance^23^. SYL3C, a DNA aptamer specifically targeting EpCAM^24,25^, has been validated for in vitro diagnostic purposes, demonstrating 92.3% sensitivity and 87.5% specificity in esophageal cancer tissue imaging^26^. Additionally, SYL3C has been successfully utilized to capture circulating tumor cells, achieving an efficiency greater than 85%. Despite these advances, most research has predominantly focused on nanoparticle-conjugated aptamer-EpCAM complexes; for instance, the EpCAM aptamer-nanoparticle complex exhibited a tumor inhibition rate of 71.3%^27^. However, the metabolic kinetics, temporal targeting properties, and in vivo imaging specificity of unmodified EpCAM-targeting aptamers remain insufficiently characterized. Importantly, investigation of the intrinsic structure-activity relationship of aptamer probes is essential to guide rational engineering and chemical modification. Elucidating the early metabolic behavior and targeting features of unmodified SYL3C probes in vivo will address a significant gap in basic research and inform the optimization of time-sensitive applications, such as intraoperative real-time navigation. At present, the absence of dynamic in vivo imaging data for SYL3C directly impedes its clinical translation.

In this study, a NIRF probe, Cy7-SYL3C, was constructed using the EpCAM-targeting aptamer SYL3C as a basis. Through multi-time-point dynamic monitoring (ranging from 5 min to 48 h) and comprehensive multidimensional validation experiments, the early targeting efficacy and metabolic characteristics of Cy7-SYL3C were elucidated for the first time. The novel aspects of this work include: first, evaluate the distribution and metabolism of Cy7-SYL3C in healthy mice, with the main metabolic pathways being the liver and kidneys; second, the demonstration of rapid targeting property of Cy7-SYL3C to tumors, evidenced by continuous dynamic observation beginning as early as 5 min post-administration; and third, robust multidimensional verification of targeting ability was achieved through competitive blocking assays and spatial colocalization analysis. The research results provided crucial benchmark data for the subsequent engineering improvement of this probe, and also expanded the application of the Cy7-SYL3C in molecular imaging. Collectively, these findings are anticipated to advance the precise diagnostic application of aptamer-based probes in CRC and to establish a framework for the development of real-time, intraoperative imaging tools.

## Materials and methods

### General

The DNA oligonucleotides, including SYL3C and Cy7-SYL3C, were synthesized by Sangon Biotech (Shanghai, China). The Cy7-SYL3C probe was constructed by conjugating the CY7 fluorophore directly to the 5’-end of the SYL3C aptamer without an additional spacer/linker. The sequence of SYL3C is as follows: 5’-CAC TAC AGA GGT TGC GTC TGT CCC ACG TTG TCA TGG GGG GTT GGC CTG-3’^38^. Roswell Park Memorial Institute (RPMI) 1640 medium and Dulbecco’s modified Eagle’s medium (DMEM) were supplied by Gibco (Grand Island, NY, USA). Cell lines were obtained from ComWin Biotech Co., Ltd. (Guangzhou, China), including the human colorectal carcinoma cell line HT-29 (ATCC^®^ CCL-185™) and the human normal colon epithelial cell line NCM460 (ATCC^®^ CRL-2654™), which were both used in this study. Chemical reagents were purchased from Beijing Solarbio Science & Technology Co., Ltd. and Beijing Biotopped Technology Co., Ltd. (Beijing, China) and were used as received unless otherwise specified. Primary and secondary antibodies for Western blotting were acquired from Guangzhou Bio-Platform Biotechnology Co., Ltd. (Guangzhou, China). The enhanced chemiluminescence (ECL) kit was purchased from Wuhan Servicebio Technology Co., Ltd. (Wuhan, China).

### In vitro stability

The Cy7-SYL3C probe was diluted in 1× phosphate-buffered saline (PBS) to a stock concentration of 10 µM. This stock solution was then mixed with 100% mouse serum, fetal bovine serum (FBS), or 1× PBS buffer at a 1:10 volume ratio. This resulted in a final probe concentration of 1 µM in each incubation mixture. The mixtures were maintained at 37 °C for 24 h. Stability was evaluated by electrophoresis on a 2% agarose gel using TAE-Mg^2+^ running buffer. Band intensities were quantified with ImageJ software.

### Cytotoxicity assay

The cytotoxicity of Cy7-SYL3C was evaluated using a Cell Counting Kit-8 (CCK-8). HT-29 cells were maintained in RPMI 1640 medium, while NCM460 cells were cultured in DMEM. Both media were supplemented with 10% FBS and 1% penicillin/streptomycin. Cells were incubated at 37 °C in a humidified atmosphere containing 5% CO_2_. Following overnight seeding of 5,000 cells per well in 96-well plates, cells were treated with varying concentrations of Cy7-SYL3C (0.2, 0.4, 0.6, 0.8, 1, and 2 µM; 10 µL/well) for 24 h at 37 °C. After treatment, 10 µL of CCK-8 solution was added to each well, and the plates were incubated for an additional 4 h at 37 °C. Absorbance was then measured at 450 nm using a microplate reader (Model RT-2100 C, Rayto Life and Analytical Sciences Co., Ltd., Shenzhen, China).

### Hemolysis assay

The hemolytic activity of Cy7-SYL3C was assessed at concentrations of 10, 50, and 100 µM. Freshly isolated mouse red blood cells (RBCs) were incubated with the probes at 37 °C for 1 h. Ultrapure water and PBS served as positive and negative controls, respectively. After incubation, samples were centrifuged at 13,000 × g for 15 min. Subsequently, 100 µL of supernatant from each well was carefully transferred to a new 96-well plate. The absorbance at 540 nm was measured using a microplate reader (Model RT-2100 C, Rayto Life and Analytical Sciences Co., Ltd., Shenzhen, China). The percentage of hemolysis was calculated using the following equation:

$$\% {\text{ }}Hemolysis=\left( {\left[ {{A_{540}}{ - _{\hbox{min} }}{A_{540}}} \right]} \right)/\left( {\left[ {_{{\hbox{max} }}{A_{540}}{ - _{\hbox{min} }}{A_{540}}} \right]} \right) \times 100\%$$

where A_540_ represents the absorbance value of the sample, _min_A_540_ corresponds to the absorbance of the PBS control, and _max_A_540_ corresponds to the absorbance of the ultrapure water control.

### In vivo safety evaluation

To assess in vivo biosafety, hematoxylin and eosin (H&E) staining was performed on major organs. Healthy BALB/c mice (6–8 weeks old, 18–22 g) were intravenously injected with Cy7-SYL3C conjugates at a dose of 3 nmol in 100 µL of PBS, while the control group received an equal volume of PBS. After 24 h, key organs including the heart, liver, spleen, lungs, kidneys, and brain were harvested, rinsed with PBS, and fixed in 4% paraformaldehyde at 4 °C. The tissues were then embedded in paraffin, sectioned, and subjected to H&E staining. Histological changes were evaluated under a light microscope (Model DM2000 LED, Leica Microsystems GmbH, Wetzlar, Germany). This in vivo safety evaluation was conducted following established practices for the toxicological assessment of molecular probes and nanomaterials^38^.

### HT-29 xenograft model

Male BALB/c nude mice (6–8 weeks old) were obtained from Beijing Vital River Laboratory Animal Technology Co., Ltd. (China). Animals were housed in the Experimental Animal Center of Inner Mongolia Medical University under specific conditions: a 12-hour light/dark cycle, an ambient temperature of 21–26 °C, and relative humidity maintained at 41–62%. Standard rodent food and purified water were provided ad libitum, and bedding was changed two to three times weekly. Mice were acclimated for 5–7 days prior to experimentation. For the establishment of the HT-29 CRC subcutaneous xenograft model, 1 × 10^6^ HT-29 cells were inoculated into the right axilla of each mouse. When the tumor volume reached 6–8 mm^3^, the mice were used for in vivo imaging and biodistribution assessments. Tumor volume (V) was calculated using the formula: V = (L × W^2^) / 2, where L is the longest diameter (length) and W is the shortest diameter (width) of the tumor. Anesthesia was induced with isoflurane gas before any potentially distressing procedures. At the end of the experiments, mice were humanely euthanized by an intraperitoneal injection of an overdose of sodium pentobarbital (150 mg/kg). Death was confirmed by the absence of a pedal reflex and cessation of respiration for more than 5 min.

All experimental protocols were approved by the Animal Ethics Committee of Inner Mongolia Medical University (Approval No. YKD202402026) and were in accordance with the NIH Guide for the Care and Use of Laboratory Animals (1996).

### Western blot validation of EpCAM expression

Proteins were extracted from HT-29 xenograft tumors as well as major organs (heart, liver, spleen, lungs, and kidneys). The protein lysates were separated by 10% SDS-PAGE and electrotransferred onto 0.45 μm PVDF membranes. After blocking with 5% skim milk for 30 min, the membranes were incubated overnight at 4 °C with an anti-EpCAM primary antibody. This was followed by incubation with a horseradish peroxidase (HRP)-conjugated secondary antibody for 30 min at room temperature. Protein bands were visualized using an ECL detection kit. Band intensities were quantified using ImageJ software.

### Immunofluorescence

The Cy7-SYL3C aptamer was dissolved in nuclease-free water to prepare a primary stock solution. For experimental use, an aliquot was diluted in 1× PBS to the desired working concentration. Two dosing strategies were employed as follows: (1) the Cy7-SYL3C group received an intravenous injection of 3 nmol Cy7-SYL3C in 100 µL PBS; (2) the pre-blocking group was first administered 15 nmol of unlabeled SYL3C in 100 µL PBS, followed immediately by 3 nmol of Cy7-SYL3C in 100 µL PBS. All injections were performed via the tail vein in HT-29 tumor-bearing BALB/c nude mice. Mice were euthanized and dissected 24 h post-injection. Tumors were harvested, fixed in 4% paraformaldehyde, dehydrated through graded ethanol series, paraffin-embedded, and sectioned. For EpCAM targeting, sections were stained with Goat Anti-Rabbit IgG H&L (FITC, Ex: 490 nm, Em: 525 nm, ab6939, Abcam, UK). Nuclear counterstaining was performed using 4’,6-diamidino-2-phenylindole(DAPI). Fluorescence imaging was conducted with a digital pathology scanner (model KF-FL-020, Jiangfeng Biotechnology Information Co., Ltd., Ningbo, China). Image analysis and processing were carried out using KFBIO Digital Slide Viewer software (version 1.7.0.21, Jiangfeng Biotechnology Information Co., Ltd.). Results were interpreted based on DAPI staining of nuclei (blue), specific EpCAM labeling (green), and the distribution of the injected fluorescent probes under ultraviolet laser excitation (pink).

### Fluorescence imaging

Cy7-SYL3C and SYL3C were prepared in PBS at the required concentrations for all experiments. Subsequently, 3 nmol Cy7-SYL3C was administered via tail vein injection to three healthy mice and three HT-29 tumor-bearing mice. Additionally, a solution containing 15 nmol SYL3C mixed with 3 nmol Cy7-SYL3C was administered to another group of HT-29 tumor-bearing mice (*n* = 3). Fluorescence imaging was conducted using an in-vivo imaging system (AniView 600, Guangzhou Biolighting Biotechnology Co., Ltd., Guangzhou, China). Mice were anesthetized with a small animal gas anesthesia system (AA-500, same manufacturer), maintaining anesthesia with 2–2.5% isoflurane throughout the imaging process. Imaging conditions were standardized as follows: camera temperature set to 21 °C, acquisition modes including both bright field and fluorescence imaging, field of view of 220 × 180 mm, exposure time of 0.16 s, and binning set to 2 × 2. For Cy7 fluorescence detection, the excitation and emission wavelengths were 730 nm and 820 nm, respectively. Mice were positioned in supine, prone, and left lateral recumbency, and imaging was performed at multiple time points post-injection: 5 min, 15 min, 30 min, 1 h, 2 h, 4 h, 6 h, 8 h, 12 h, 24 h, and 48 h. At 48 h after injection, the major organs, including the heart, liver, spleen, lungs, kidneys, as well as tumor tissues, were harvested for ex vivo fluorescence imaging. Regions of interest (ROIs) corresponding to the liver, kidneys and tumors were delineated for semi-quantitative analyses. Throughout the entire 48-hour period, the mice had ad libitum access to food and water. They were maintained under isoflurane anesthesia only during the brief image acquisition sessions at each time point.

### Statistical analysis

All data were analyzed using GraphPad Prism software (version 10.0). Quantitative results are presented as mean ± standard deviation (SD). Statistical differences among groups were evaluated by one-way analysis of variance (ANOVA). A p-value less than 0.05 was considered to indicate statistical significance. The sample size (*n* = 3 per group) was chosen based on established conventions for preliminary feasibility studies in preclinical optical imaging^29^, where the objective is to detect large effect sizes and promising trends prior to larger-scale validation. While this sample size limits the power to detect more subtle differences, the statistically significant results obtained in primary outcomes (e.g., tumor uptake) indicate that the observed effects are substantial.

## Results

### In vitro stability and biosafety assessment of Cy7-SYL3C

**Fig. 1:**
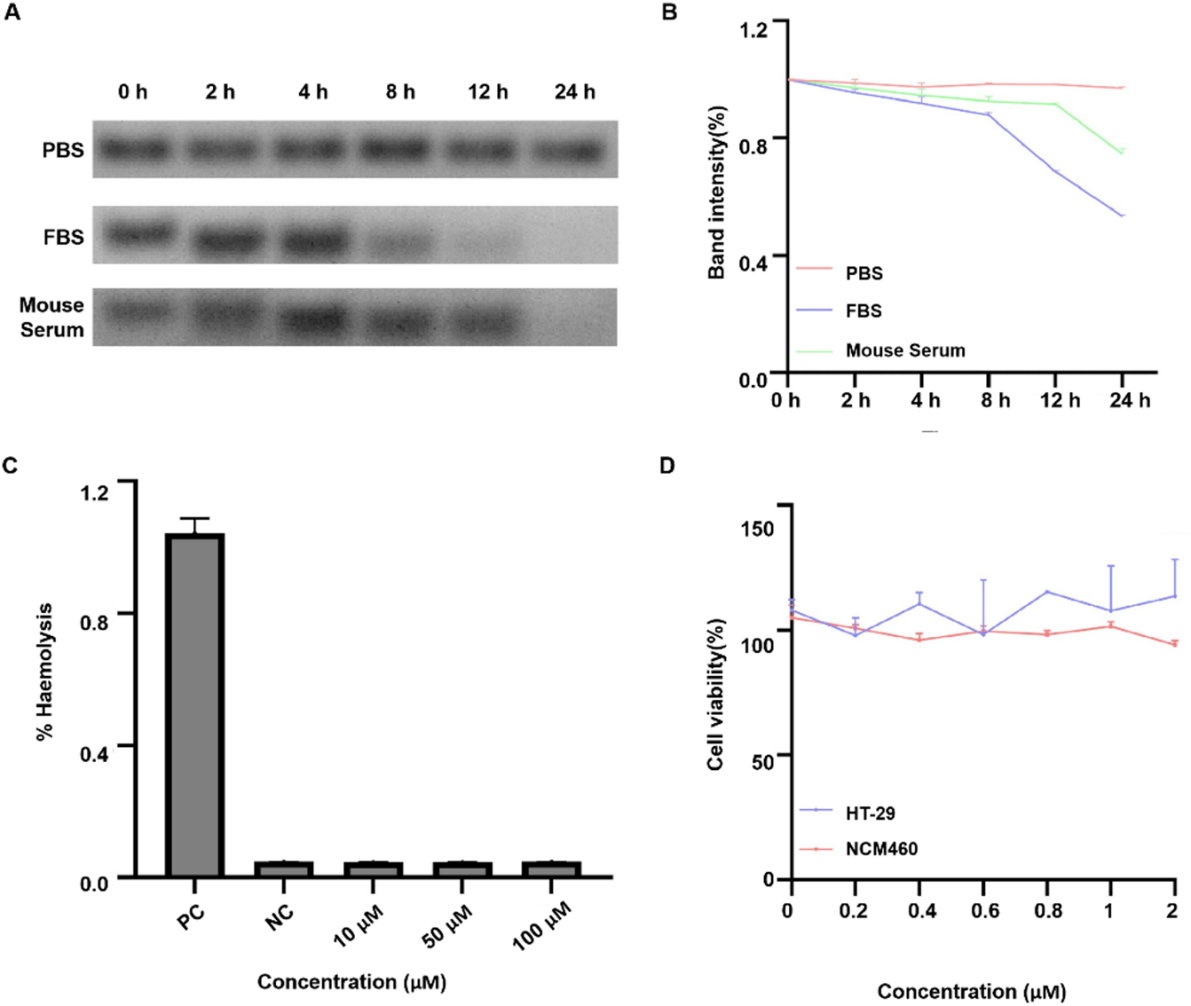
In vitro stability and biosafety evaluation of Cy7-SYL3C. (**A**) Assessment of Cy7-SYL3C stability in PBS, 100% FBS, and 100% mouse serum by 2% agarose gel electrophoresis. (**B**) Quantitative densitometric analysis of band intensity from (**A**), performed using ImageJ software. (**C**) Measurement of erythrocyte hemolysis rates induced by escalating concentrations of Cy7-SYL3C (50–100 µM), with ultrapure water and PBS serving as positive and negative controls, respectively. (**D**) Cytotoxicity evaluation of Cy7-SYL3C on HT-29 and NCM460 cells following 24 h incubation, determined by the CCK-8 assay.

To evaluate the in vitro stability of Cy7-SYL3C, the compound was incubated in PBS, 100% FBS, and 100% mouse serum. The analysis demonstrated that approximately 80% of Cy7-SYL3C maintained its structural integrity in both mouse serum and FBS after 8 h of incubation (Fig. 1A,B). Cytotoxicity was investigated using the CCK-8 assay: after exposure of HT-29 and NCM460 cells to varying concentrations of Cy7-SYL3C for 24 h, cell viability remained largely unaffected within the 0.2 to 2.0 µM concentration range (Fig. 1C). Furthermore, the hemolytic potential of Cy7-SYL3C was assessed at a concentration of 2 µM and found to be negligible, displaying nonhemolytic characteristics (Fig. 1D). Hemolysis values below 5% were not considered significant, in accordance with established safety standards for hemocompatibility assessment^30^. Collectively, these results indicate that Cy7-SYL3C exhibits favorable biocompatibility and stability, making it a suitable candidate for further in vivo investigations.

### Biodistribution of Cy7-SYL3C in healthy mice

**Fig. 2:**
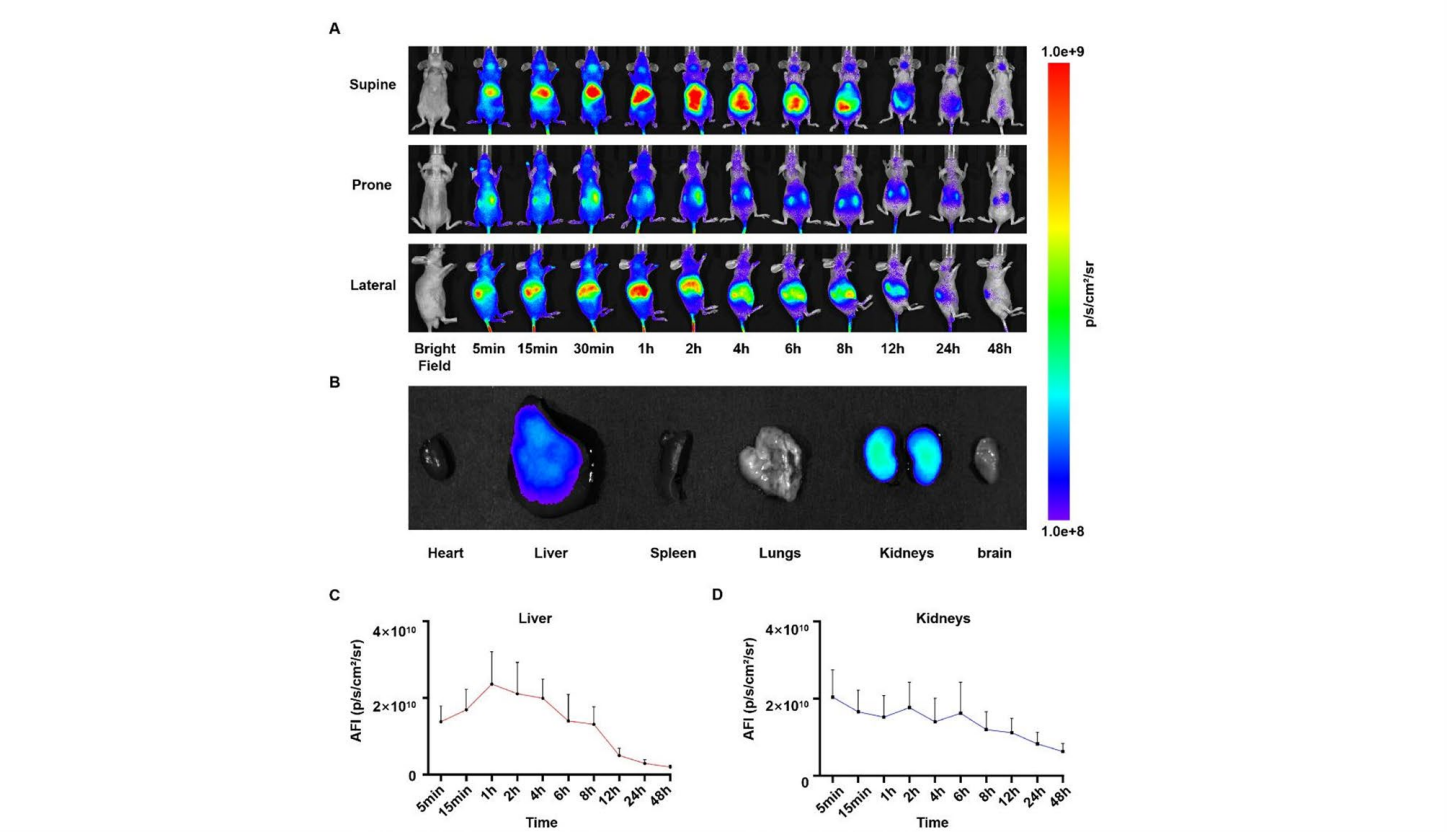
The biodistribution, organ accumulation, and quantitative analysis of Cy7-SYL3C in healthy mice. (**A**) Sequential in vivo fluorescence imaging of healthy mice following intravenous administration of Cy7-SYL3C at various time points (5 min, 15 min, 30 min, 1 h, 2 h, 4 h, 8 h, 12 h, 24 h, and 48 h; *n* = 3). (**B**) Ex vivo fluorescence imaging of major organs (heart, liver, spleen, lungs, kidneys, and brain) collected 48 h after injection. (**C**) Quantification of AFI of Cy7-SYL3C in the liver at different time points post-injection. (**D**) Quantification of AFI of Cy7-SYL3C in the kidneys at corresponding time points post-injection.

**Fig. 3:**
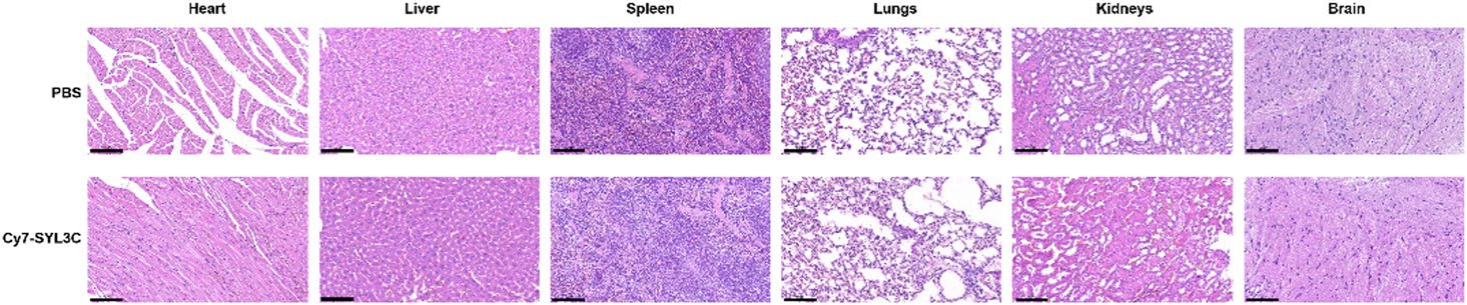
Histological evaluation of major organs in healthy mice. Representative hematoxylin and eosin (H&E) stained sections of the heart, liver, spleen, lungs, kidneys, and brain harvested 48 h after administration of Cy7-SYL3C or PBS (control). Scale bars = 100 μm.

Healthy BALB/c mice (*n* = 3) were intravenously injected with 3 nmol of Cy7-SYL3C in 100 µL of PBS via the tail vein. Under 2% isoflurane anesthesia, serial in vivo fluorescence imaging was performed using a SAFI system (AniView 600) at multiple time points (5 min, 15 min, 30 min, 1 h, 2 h, 4 h, 8 h, 12 h, 24 h, and 48 h), with the mice positioned supine, prone, and in left lateral recumbency (Fig. 2A). Quantitative analysis demonstrated that Cy7-SYL3C was primarily distributed in the liver and kidneys during the first 4 hours post-injection, with the highest liver signal intensity observed at 1 h (1.2 ± 0.2) × 10^5^ photons/s/mm^2^. Subsequently, the fluorescence signal became increasingly localized to the kidneys, reaching a signal intensity of (8.7 ± 0.9) × 10^4^ photons/s/mm^2^ at 24 h. By 48 h, the liver signal diminished to 12.3% ± 2.1% of its peak value (*p* < 0.001). The significant and sustained accumulation of the probe in the kidneys, evidenced by a signal intensity of (8.7 ± 0.9) × 10^4^ p/s/mm^2^ at 24 h, supports that renal clearance is a major elimination pathway for Cy7-SYL3C (Fig. 2B,C). The rapid elimination profile (t_1/2_ = 6.5 ± 0.7 h) may be attributed to the low molecular weight of Cy7-SYL3C (14.8 kDa). Furthermore, histological examination using H&E staining revealed no significant morphological alterations or apparent tissue damage in major organs when comparing the Cy7-SYL3C group with the PBS control group (Fig. 3).

### Verification of Cy7-SYL3C binding to EpCAM

**Fig. 4:**
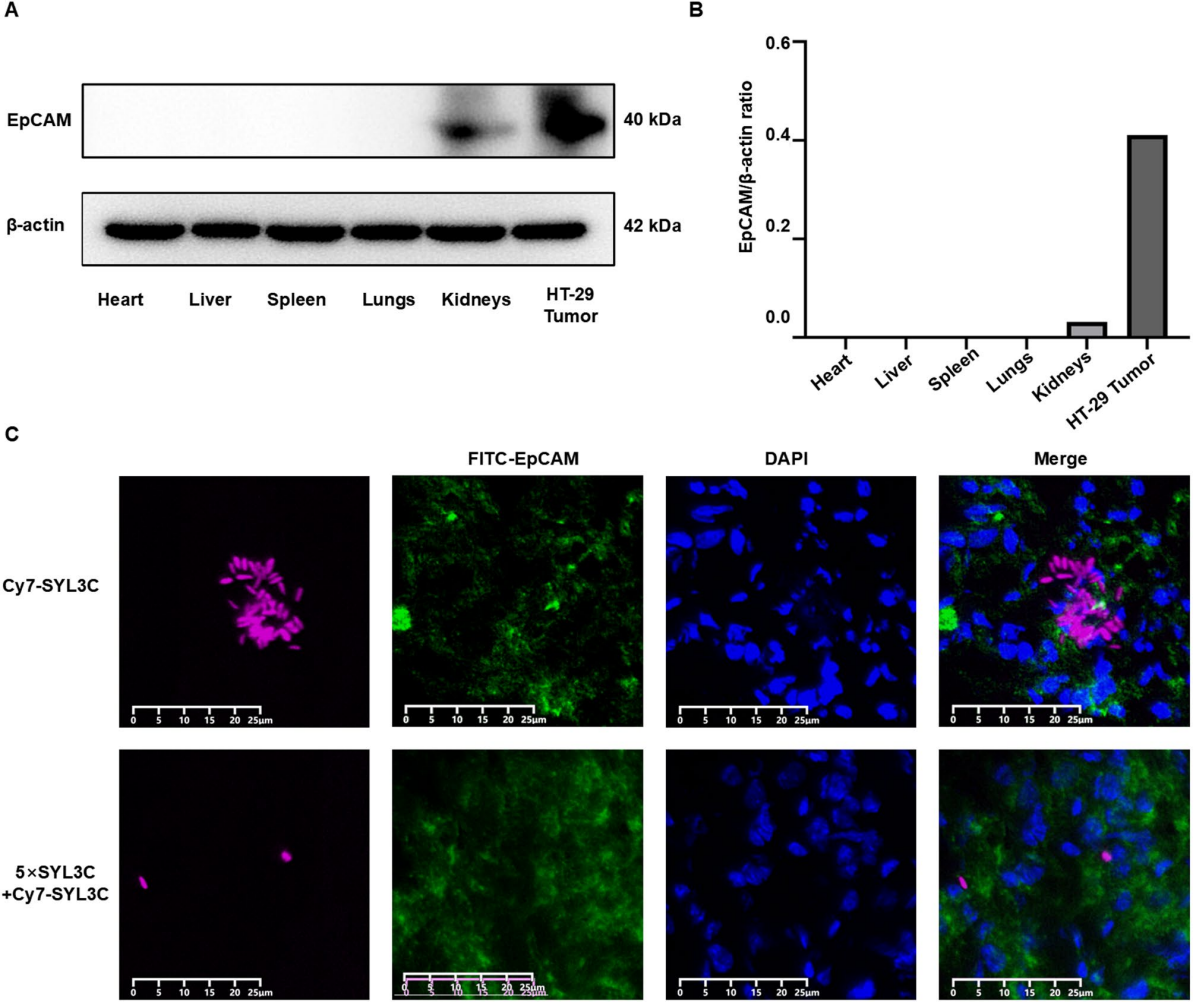
Validation of EpCAM expression and binding affinity of Cy7-SYL3C to EpCAM. (**A**) Western blot analysis demonstrates EpCAM expression in HT-29 tumors compared to major organs (heart, liver, spleen, lungs, and kidneys) from tumor-bearing mice, performed using 10% SDS-PAGE and β-actin as the internal control. (**B**) Quantitative densitometric analysis of Western blot bands using ImageJ software, with results expressed as the EpCAM/β-actin ratio. (**C**) Ex vivo immunofluorescence imaging of HT-29 tumor-bearing mice 24 h after injection of 1 nM Cy7-SYL3C alone or in combination with a five-fold molar excess of SYL3C. Scale bar = 25 μm. The Cy7 fluorophore (excitation: 749 nm; emission: 820 nm) labels SYL3C and appears as magenta fluorescence. FITC (excitation: 490 nm; emission: 525 nm) marks the EpCAM target on the HT-29 cell surface, visualized as green fluorescence. DAPI (excitation: 330–380 nm; emission: 420 nm) stains the nucleus and is shown as blue fluorescence. Merged images reveal the co-localization of all fluorescent tags. Fluorescence images were acquired using a digital pathology scanner (model KF-FL-020, Jiangfeng Biotechnology Information Co., Ltd., Ningbo, China) and processed with KFBIO Digital Slide Viewer software (version 1.7.0.21, Jiangfeng Biotechnology Information Co., Ltd.).

Tumor tissues and major organs (heart, liver, spleen, lungs, and kidneys) were collected from HT-29 tumor-bearing nude mice for Western blot analysis. The results showed robust EpCAM expression in HT-29 tumors, minimal expression in kidneys, and no detectable expression in other organs. β-actin served as the loading control (Fig. 4A,B). For in vivo targeting validation, HT-29 tumor-bearing mice (*n* = 3 per group) were intravenously injected via the tail vein with the following: (1) Cy7-SYL3C group: 1 nM Cy7-SYL3C; (2) Pre-blocking group: 5 nM unlabeled SYL3C administered prior to 1 nM Cy7-SYL3C. Tumor sections were analyzed by immunofluorescence 24 h post-injection, revealing moderate colocalization of Cy7-SYL3C (pink) and FITC-anti-EpCAM (green) in the Cy7-SYL3C group. In contrast, the pre-blocking group exhibited only minimal and scattered fluorescence signals (Fig. 4C). Quantitative co-localization analysis was performed using the JACoB plugin of ImageJ software. The findings indicated that the Pearson’s coefficients of the Cy7-SYL3C group was 0.30 ± 0.02, suggesting a modest yet statistically significant positive correlation between the fluorescence signal and EpCAM expression. In contrast, the correlation coefficient of the pre-blockade group was only 0.05 ± 0.01, indicating an almost negligible correlation between the two variables. This result further confirmed that the pre-saturation treatment without labeling SYL3C significantly reduced the specific binding of Cy7-SYL3C to the target EpCAM, thereby effectively blocking its targeting recognition ability.

### Tumor-targeted fluorescence imaging in HT-29 xenograft models

**Fig. 5:**
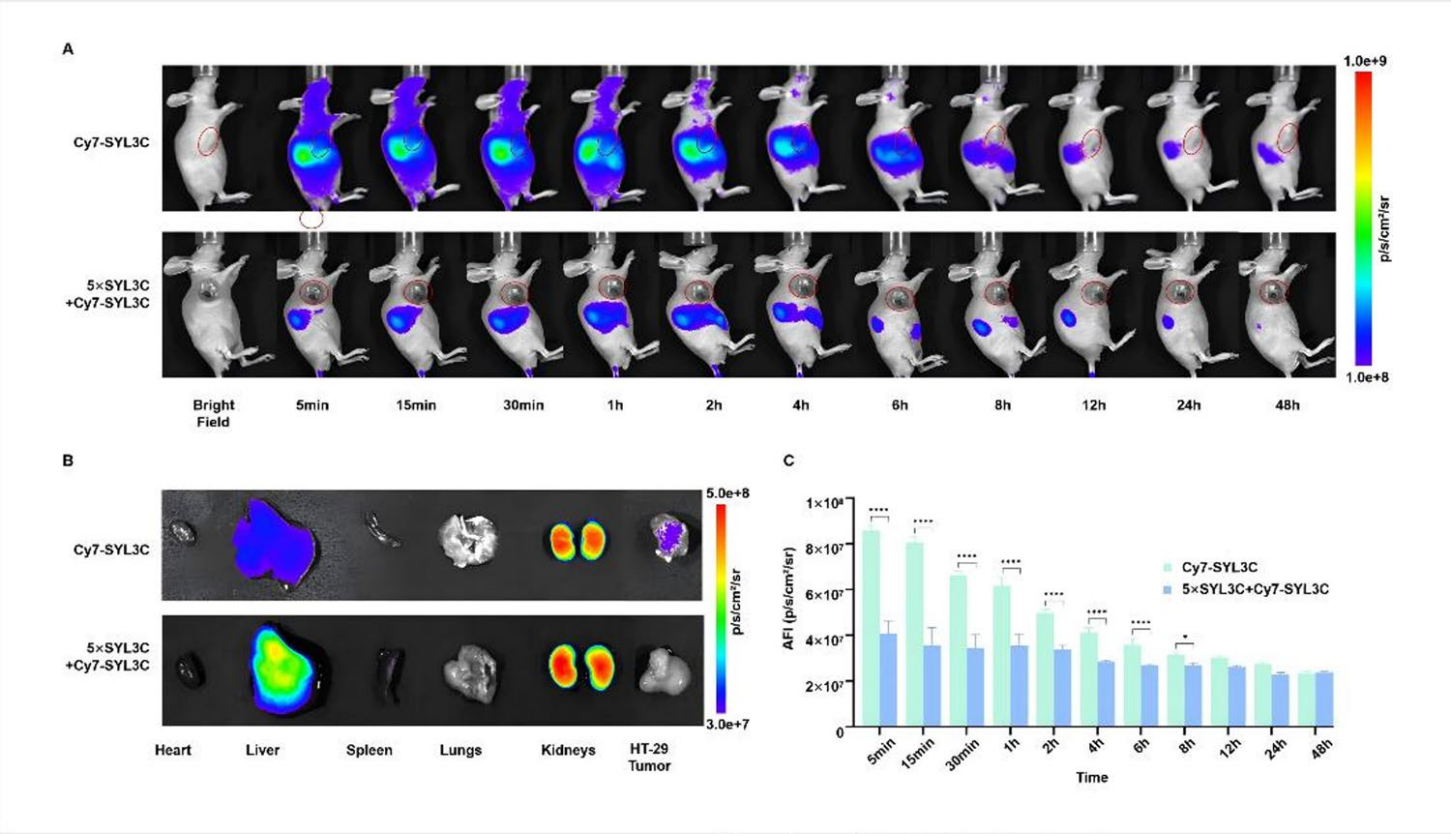
The biodistribution, organ accumulation, and tumor uptake quantification of Cy7-SYL3C in HT-29 tumor-bearing mice, including competitive inhibition analysis. (**A**) Sequential in vivo fluorescence imaging of mice after intravenous administration of either 1 nM Cy7-SYL3C alone (Cy7-SYL3C group) or pre-injection with 5 nM unlabeled SYL3C followed by 1 nM Cy7-SYL3C (pre-blocking group), with imaging performed at 5 min, 15 min, 30 min, 1 h, 2 h, 4 h, 8 h, 12 h, 24 h, and 48 h (*n* = 3). Tumor locations are indicated by red circles. (**B**) Ex vivo fluorescence imaging of harvested organs (heart, liver, spleen, lungs, kidneys) and tumors 48 h post-injection. (**C**) Quantification analysis of AFI in the tumor region at various time points (*n* = 3). Data are presented as mean ± SD; *P < 0.05, **P < 0.01, ***P < 0.001, ****P < 0.0001 (comparison between Cy7-SYL3C group and pre-blocking group at each time point).

HT-29 tumor-bearing mice (*n* = 3 per group) were intravenously injected with the following: the Cy7-SYL3C group received 3 nmol of Cy7-SYL3C in 100 µL of PBS, whereas the pre-blocking group received a combination of 15 nmol unlabeled SYL3C and 3 nmol Cy7-SYL3C, each in 100 µL of PBS. Serial in vivo fluorescence imaging was conducted under 2% isoflurane anesthesia from 5 min to 48 h post-injection, demonstrating rapid tumor targeting with detectable specific signals as early as five minutes post-administration (Fig. 5A). Quantitative analysis revealed that the average fluorescence intensity (AFI) at the tumor site in the experimental group was 88.2% higher than that of the pre-blocking group during the 4-hour observation period (6.4 × 10^7^ photons/s/mm^2^ vs. 3.4 × 10^7^ photons/s/mm^2^, respectively). The tumor-to-muscle ratio (T/M) increased over time, reaching 1.15 ± 0.05 at 4 h, and peaking at 1.30 ± 0.04 at 8 h post-injection, before gradually declining to 1.10 ± 0.03 by 24 h. This quantitative profile underscores the specific accumulation and prolonged retention of Cy7-SYL3C in the tumor tissue. This indicates that the EpCAM-targeting effect mediates the long-term retention of the drug at the tumor site. Forty-eight hours following injection, ex vivo fluorescence imaging of excised tumors and major organs (heart, liver, spleen, lungs, and kidneys) demonstrated preferential accumulation of Cy7-SYL3C in tumor tissue (Fig. 5B,C), in alignment with the specific binding observed in immunofluorescence studies.

## Discussion

In this study, we successfully developed the NIRF probe Cy7-SYL3C, which specifically targets EpCAM. Cy7-SYL3C exhibited remarkable stability in a serum environment, retaining over 80% of its structural integrity after 8 h of incubation (Fig. 1A,B). The progressive upward shift of the bands over time is indicative of nuclease-mediated hydrolysis of the aptamer backbone, generating lower molecular weight oligonucleotide fragments. Although transient interactions with serum proteins (such as albumin) could potentially contribute to the observed band shift phenomena, the electrophoretic pattern is not consistent with the formation of stable, high-order protein-aptamer complexes, for which no evidence was observed. Given that oligonucleotide-protein binding can significantly influence a probe’s circulation half-life and biodistribution, future studies specifically designed to quantify the affinity of Cy7-SYL3C for serum albumin will be crucial to fully elucidate its pharmacokinetic profile. Importantly, no cytotoxicity or hemolytic activity was detected across the concentration range of 0.2–2.0 µM (Fig. 1C, D), underscoring its excellent biocompatibility. The in vivo imaging and tissue distribution profile of Cy7-SYL3C delineated its targeting efficiency and clearance pathways, characteristic of a low-molecular-weight agent. Upon intravenous administration, the probe rapidly distributed to both the liver and kidneys, reaching a peak liver signal at one hour (1.2 ± 0.2) × 10^5^ photons/s/mm^2^, and was efficiently cleared renally (t₁_/_₂ = 6.5 ± 0.7 h; residual signal decreased to 12.3% ± 2.1% after 48 h) (Fig. 2). These findings align with the well-established concept that renal filtration predominates for probes with a molecular weight below 30 kDa^31,32^. The probe’s rapid clearance contributed to a significant reduction in background signal during intraoperative imaging, thereby providing a robust technical foundation for real-time tumor visualization. Furthermore, histopathological examination did not reveal any evidence of organ toxicity or damage (Fig. 3). It is noteworthy that the Cy7 fluorophore in Cy7-SYL3C is directly conjugated to the aptamer without an intervening linker, a design consistent with the original reporting of the SYL3C aptamer. While this suggests that the observed binding affinity is representative for this specific molecular construct, it is important to acknowledge that the choice and mode of fluorophore attachment can inherently influence aptamer folding and target interaction. Therefore, the measured affinity should be interpreted within the context of this direct conjugation scheme. Future optimization could systematically explore the effect of different linkers or fluorophore types on probe performance. An important methodological consideration is that the binding assays in this study were conducted without applying the specific thermal restructuring protocol (denaturation at 95 °C followed by rapid cooling on ice) described in the original SELEX procedure for the SYL3C aptamer^38^. This step is designed to ensure a uniform, energetically favorable conformation across the aptamer population. Its omission may result in a mixture of folded and misfolded structures, potentially leading to an underestimation of the true binding affinity. Therefore, the affinity values reported here should be interpreted within the context of this simplified folding protocol. Future studies will rigorously incorporate this thermal conditioning step to obtain a more accurate assessment of the aptamer’s intrinsic binding potential.

Based on our previous research foundation^33^, this study further verified the targeting specificity of the Cy7-SYL3C probe by increasing the imaging time points in live animals, investigating the distribution characteristics in healthy mice, and combining with competitive binding experiments. In vivo imaging demonstrated its time-dependent accumulation within HT-29 xenografts. Western blot analysis revealed abundant EpCAM expression in HT-29 tumor tissues, with negligible expression detected in normal organs (Fig. 4A,B). It is noteworthy that a moderate increase in liver signal was observed in the pre-blocking group in comparison to the experimental group. This phenomenon is hypothesised to be attributable to the saturation of EpCAM target sites, which results in the redirection of a greater proportion of the circulating probe towards non-specific clearance by the mononuclear phagocyte system in the liver. In pre-blocking experiments, pre-administration of unlabeled SYL3C resulted in an 88.2% reduction in tumor signal intensity (Fig. 5). Furthermore, immunofluorescence co-localization analysis demonstrated a significant decrease in the Pearson correlation coefficient from 0.30 to 0.05 (Fig. 4C), further corroborating the specificity of SYL3C for EpCAM, as supported by existing literature^34^. The probe showed persistent accumulation at the tumor site, with a tumor-to-muscle signal ratio exceeding 1.30 at 6 h post-injection, indicating tumor targeting and favorable retention characteristics. This prolonged and retained fluorescent signal at the tumor site provides sufficient time for the delineation of the tumor margin during the operation. While the specific tumor uptake, combined with the effective blocking of the signal in vivo, provides functional evidence for EpCAM-specific targeting, future studies incorporating in vitro cellular binding and internalization assays (e.g., using flow cytometry with EpCAM-positive and EpCAM-negative cell lines) will be essential to quantitatively determine binding affinity and elucidate the precise cellular kinetics of Cy7-SYL3C.

Compared to antibody-based detection modalities such as EpCAM antibody-mediated magnetic bead separation for circulating tumor cells^35^, Cy7-SYL3C offers several distinct advantages. These include a significantly lower molecular weight (14.8 kDa vs. the typical 150 kDa for full antibodies), more rapid tumor accumulation (signal peaks at 5 min post-injection versus several hours for antibody probes), and a prolonged diagnostic window (sustained tumor-to-background ratio > 1.3 for up to 6 h). The in vivo behavior of Cy7-SYL3C presents a distinct profile when systematically compared with other EpCAM-targeting probes. While antibody-based agents typically require extended circulation periods (> 24 h) to achieve optimal imaging contrast due to their prolonged blood persistence, Cy7-SYL3C establishes effective tumor-to-background differentiation within a substantially shorter timeframe (within 8 h), attributable to its rapid distribution and elimination profile. In contrast to nanoparticle systems that predominantly rely on passive accumulation mechanisms, the minimal steric hindrance of Cy7-SYL3C enables more efficient target accessibility and tissue diffusion. This combination of rapid targeting establishment, high specificity, and prompt clearance defines Cy7-SYL3C as particularly suitable for time-sensitive intraoperative imaging applications. It should be noted that the rapid blood clearance of the probe limited our ability to calculate reliable tumor-to-blood ratios at most time points, which is a common characteristic of oligonucleotide-based agents. The judicious equilibrium of rapid targeting, high specificity, and efficient clearance positions Cy7-SYL3C as a favourable agent for rapid intraoperative imaging applications where time is a critical factor. Collectively, these features facilitate rapid intraoperative tumor delineation and provide real-time guidance to support precise surgical resection^36,37^. Notably, Cy7-SYL3C overcomes the slow clearance limitations associated with traditional antibody-based imaging probes and achieves enhanced tissue penetration. Moving forward, future research will be directed towards (1) optimizing pharmacokinetic parameters to further extend tumor retention, (2) investigating multimodal labeling strategies (such as incorporating radionuclides) to expand theranostic applications, and (3) conducting preclinical studies for detecting metastatic lesions.

## Conclusion

This study demonstrates that Cy7-SYL3C specifically targets EpCAM-positive CRC. The probe’s distinctive targeting and clearance properties—including rapid tumor accumulation and a dual clearance mechanism primarily via the kidneys, with additional hepatic uptake—lay a solid foundation for its potential clinical translation. Collectively, these results underscore the promise of Cy7-SYL3C as a novel molecular tool for establishing precise diagnostic and therapeutic systems in CRC, with considerable translational value.

## Supplementary Information

Below is the link to the electronic supplementary material.

Supplementary Material 1

Supplementary Material 2

## Data Availability

The original contributions presented in the study are included in the article, further inquiries can be directed to the corresponding authors.

## Abbreviations

EpCAM: Epithelial cell adhesion molecule
CRC: Colorectal cancer
NIRF: Near-infrared fluorescence
SAFI: Small animal fluorescence imaging
AFI: Average fluorescence intensity
SELEX: Systematic evolution of ligands by exponential enrichment
RPMI: Roswell Park Memorial Institute
DMEM: Dulbecco’s modified Eagle’s medium
ECL: Enhanced chemiluminescence
FBS: Fetal bovine serum
PBS: Phosphate-buffered saline
CCK-8: Cell Counting Kit-8
nm: Nanometers
RBCs: Red blood cells
H&E: Hematoxylin and eosin
HRP: Horseradish peroxidase
FITC: Fluorescein isothiocyanate
ROIs: Regions of interest
SD: Standard deviation
PC: Positive Control
NC: Negative Control
