# Supplementary material for: Preliminary assessment of biodistribution and targeting of the fluorescent molecular probe Cy7-SYL3C in an EpCAM-positive colorectal cancer mouse model
Source: Sci Rep. 2026 Jan 29;16:6589. doi: 10.1038/s41598-026-37787-2 (PMC12913630; doi:10.1038/s41598-026-37787-2)
Supplement: Supplementary file 1 — Supplementary Material 1 [file 41598_2026_37787_MOESM1_ESM.docx]

Supplementary

**Supplementary Figure 1.** The three full-length, original blots shown here correspond, from top to bottom, to the three lanes presented in Figure 1A (PBS, 100% FBS, and 100% mouse serum, respectively). These are the original, unprocessed images.

**
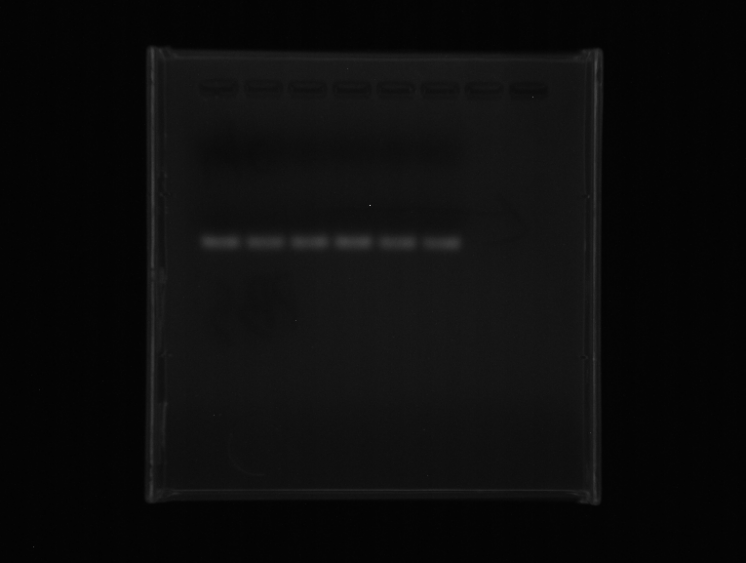
**

**PBS(The incubation times from left to right were 0h、2h、4h、8h、12h、24h)**

**
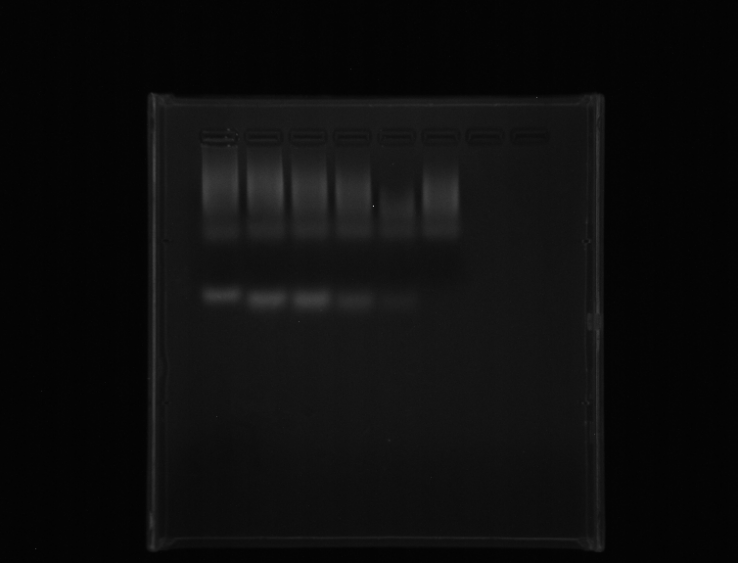
**

**FBS(The incubation times from left to right were 0h、2h、4h、8h、12h、24h)**

**
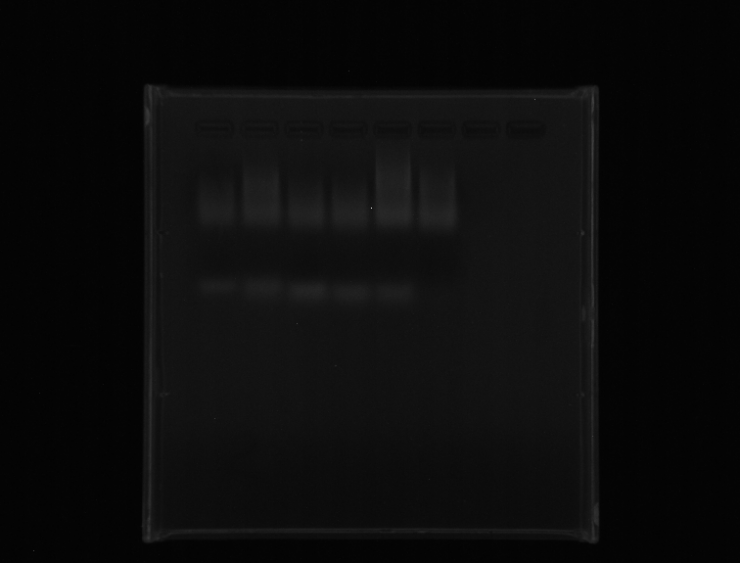
**

**100% mouse serum(The incubation times from left to right were 0h、2h、4h、8h、12h、24h)**

**Supplementary Figure 2.** Uncropped blots corresponding to Figure 4A. The two full-length, original blots shown here correspond, from top to bottom, to the two lanes presented in Figure 4A (HT-29 tumor and major organs, respectively). These are the original, unprocessed images.

**
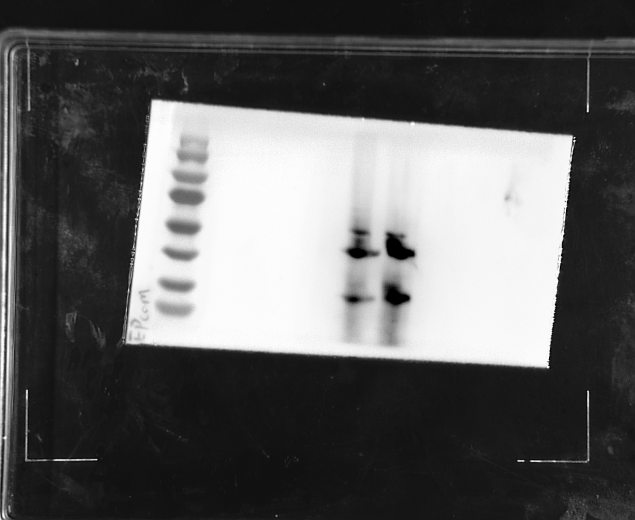
**

**EpCAM(The tissues being examined are arranged from left to right as follows:maker、Heart、Liver、Spleen、Lungs、Kidneys、HT-29 Tumor)**

**
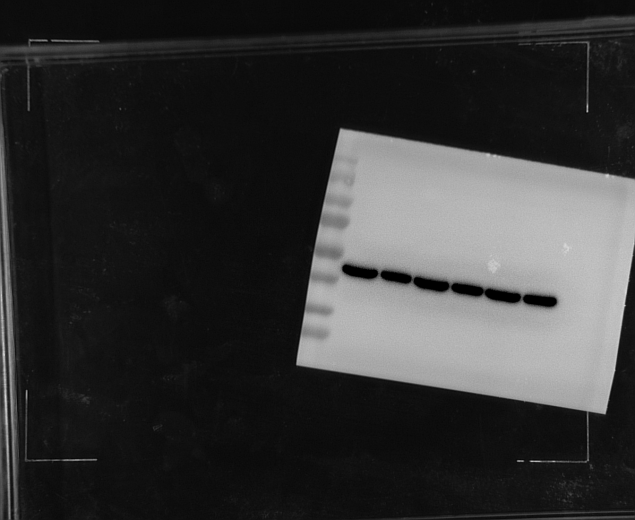
**

**β-actin(The tissues being examined are arranged from left to right as follows:maker、Heart、Liver、Spleen、Lungs、Kidneys、HT-29 Tumor)**
