# Supplementary material for: Preliminary assessment of biodistribution and targeting of the fluorescent molecular probe Cy7-SYL3C in an EpCAM-positive colorectal cancer mouse model
Source: Sci Rep. 2026 Jan 29;16:6589. doi: 10.1038/s41598-026-37787-2 (PMC12913630; doi:10.1038/s41598-026-37787-2)
Supplement: Supplementary file 2 — Supplementary Material 2 [file 41598_2026_37787_MOESM2_ESM.docx]

Supplementary materials

**Supplementary Dataset S1： Individual Longitudinal Tumor Data**

**Description:** This dataset contains longitudinal tumor measurement data and weight data for one representative mouse from the "Cy7-SYL3C group" and another from the "5×SYL3C + Cy7-SYL3C group". During the experiment, the average tumor volume was approximately 6.35 cubic millimeters.

| **Group** | **Body Weight (g)** | **Tumor Length (mm)** | **Tumor Width (mm)** | **Calculated Volume (mm³)** | **Tumor Weight (g)** |
| --- | --- | --- | --- | --- | --- |
| **Cy7-SYL3C** | **18.3** | **3.7** | **1.8** | **6.0** | **0.065** |
| **5×SYL3C+Cy7-SYL3C** | **18.5** | **2.3** | **2.4** | **6.6** | **0.075** |

**Supplementary Fig. S2: Full-length blot for Figure 1A**

**
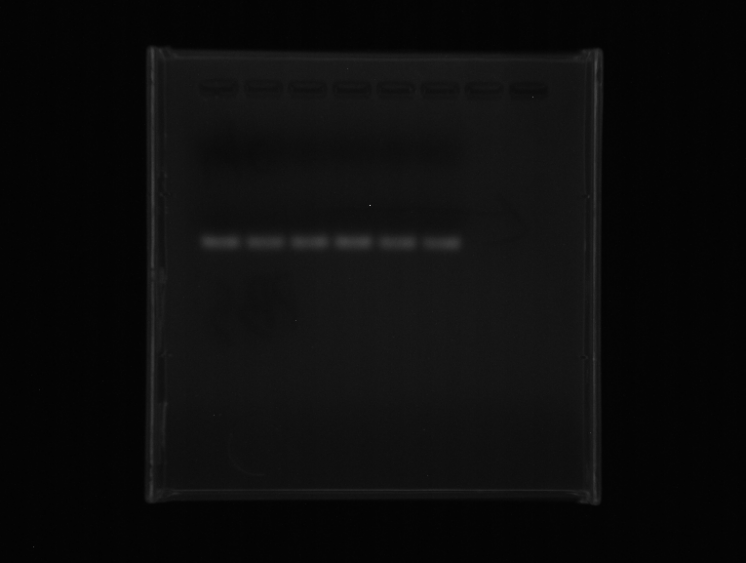
**

**Cy7-SYL3C was incubated in PBS(The incubation times from left to right were 0h、2h、4h、8h、12h、24h)**

**
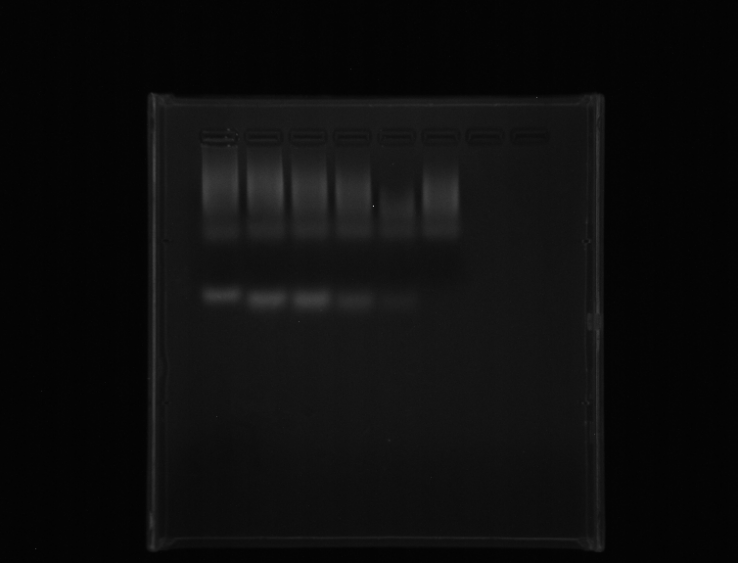
**

**Cy7-SYL3C was incubated in FBS(The incubation times from left to right were 0h、2h、4h、8h、12h、24h) The observed upward shift of the bands over time is indicative of nuclease-mediated degradation, generating oligonucleotide fragments with lower molecular weights and faster gel mobility.**

**
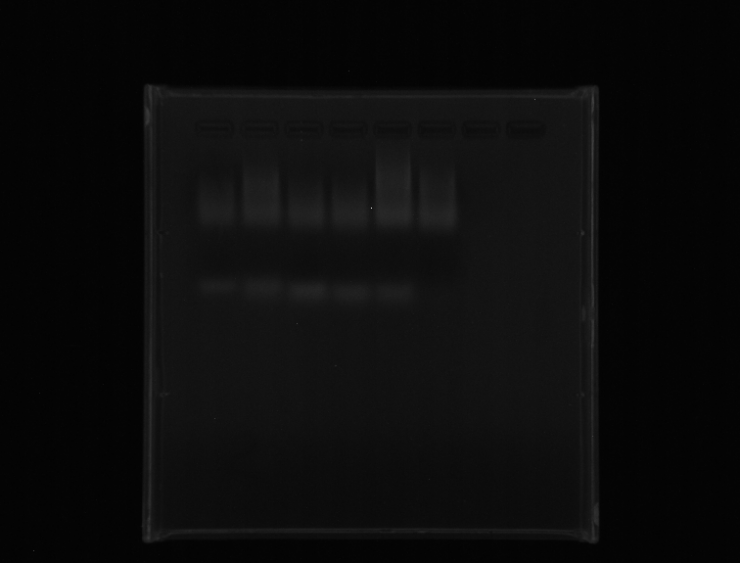
**

**Cy7-SYL3C was incubated in 100% mouse serum(The incubation times from left to right were 0h、2h、4h、8h、12h、24h) The observed upward shift of the bands over time is indicative of nuclease-mediated degradation, generating oligonucleotide fragments with lower molecular weights and faster gel mobility.**

**Supplementary Fig. S3: Full-length blot for Figure 4A**

**
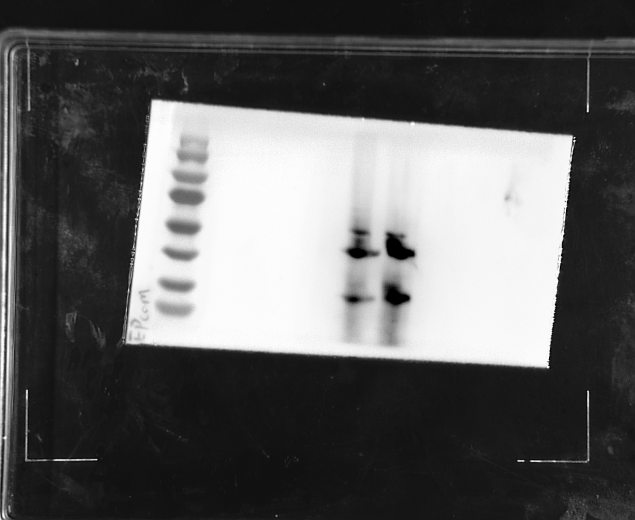
**

**EpCAM(The tissues being examined are arranged from left to right as follows:maker、Heart、Liver、Spleen、Lungs、Kidneys、HT-29 Tumor)**

**
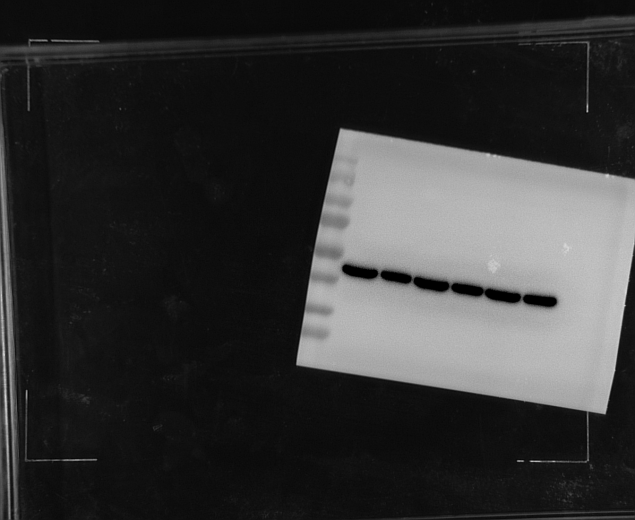
**

**β-actin(The tissues being examined are arranged from left to right as follows:maker、Heart、Liver、Spleen、Lungs、Kidneys、HT-29 Tumor)**
